# The Application of Augmented Reality Technology in Perioperative Visual Guidance: Technological Advances and Innovation Challenges

**DOI:** 10.3390/s24227363

**Published:** 2024-11-19

**Authors:** Yichun Shen, Shuyi Wang, Yuhan Shen, Jingyi Hu

**Affiliations:** School of Health Science and Engineering, University of Shanghai for Science and Technology, Shanghai 200093, China; 223332642@st.usst.edu.cn (Y.S.); 223332604@st.usst.edu.cn (Y.S.); 232322248@st.usst.edu.cn (J.H.)

**Keywords:** augmented reality technology, image processing, machine learning, perioperative visual guidance technology

## Abstract

In contemporary medical practice, perioperative visual guidance technology has become a critical element in enhancing the precision and safety of surgical procedures. This study provides a comprehensive review of the advancements in the application of Augmented Reality (AR) technology for perioperative visual guidance. This review begins with a retrospective look at the evolution of AR technology, including its initial applications in neurosurgery. It then delves into the technical challenges that AR faces in areas such as image processing, 3D reconstruction, spatial localization, and registration, underscoring the importance of improving the accuracy of AR systems and ensuring their stability and consistency in clinical use. Finally, the review looks forward to how AR technology could be further facilitated in medical applications with the integration of cutting-edge technologies like skin electronic devices and how the incorporation of machine learning could significantly enhance the accuracy of AR visual systems. As technology continues to advance, there is ample reason to believe that AR will be seamlessly integrated into medical practice, ushering the healthcare field into a new “Golden Age”.

## 1. Introduction

In modern medicine, the success of surgical operations depends not only on the surgery itself but also on scientifically effective perioperative management. As an interdisciplinary and comprehensive management discipline, the development of perioperative management has reflected a shift from a focus on single surgical techniques to a patient-centered holistic treatment approach [1]. Innovative technologies developed around perioperative management, such as digital healthcare, remote monitoring, and wearable devices, are propelling the field of health engineering towards a more scientific, systematic, and humanized direction [2,3]. Visual guidance technology plays a crucial role in surgery. It provides real-time imaging information to doctors. This information assists in better identifying and locating the surgical area during the operation. By doing so, it helps to reduce damage to surrounding tissues. Additionally, it improves surgical outcomes [4,5]. This technology is especially important in minimally invasive surgery [6] and precision medicine [7]. In these fields, its role is particularly prominent. However, traditional surgeries rely on the doctor’s experience and two-dimensional image assistance, which has certain limitations when dealing with complex or minimally invasive surgeries [8]. In contrast, Augmented Reality (AR) technology, by superimposing virtual information on the patient’s actual anatomical structure, provides doctors with a more intuitive and precise surgical navigation tool, greatly enhancing the success rate and safety of surgeries [9].

It is widely acknowledged that the origins of AR can be traced back to the 1950s, initially conceptualized by Morton Heilig [10]. The technology advanced in the 1960s with Ivan Sutherland’s invention of the head-mounted display (HMD) [11], which remains a prevalent format for presenting AR imagery to this day.

The first concrete application of Augmented Reality (AR) technology dates back to 1969 when Wright-Patterson Air Force Base in Ohio developed a night flight assistance system based on AR technology [12]. This system, equipped with sensors installed on the aircraft, enhanced the pilot’s visibility of the real world under adverse conditions such as obstructions by the aircraft’s structure or insufficient lighting. It superimposed flight data and target information onto the pilot’s field of view and provided audio cues to assist with orientation. This innovation not only improved the safety of night flights but also laid the groundwork for the development of AR technology in the future. However, it was not until 1997 that a clear technical definition of AR was provided by Azuma and others in their seminal AR survey report [13]. AR is defined as the integration of real and virtual environments, registered in 3D and interacting in real time, a definition that is now widely accepted.

The field of neurosurgery was among the pioneers in attempting to incorporate AR for visual guidance during the perioperative period. As early as 1998, Masutani and colleagues published a study on an AR visualization system to support endovascular neurosurgery [14,15]. This system reconstructed blood vessel models based on X-ray images, allowing surgeons to simultaneously view real catheters and reconstructed vascular models on a display monitor. Although the system still had limitations in the accuracy of model registration and image clarity, it offered a novel approach to perioperative visual guidance that included preoperative path planning and intraoperative real-time navigation.

Over the past two decades, perioperative visual guidance based on AR technology has undergone significant transformation, with mature solutions providing substantial support for surgical procedures. The application of AR technology in perioperative management has evolved from initial concept validation to a mature clinical tool. The development of this technology has not only enhanced the precision and safety of surgeries but also improved patient outcomes. However, despite the notable advancements in the application of AR technology in surgical procedures, the field is still rapidly evolving, with new technologies and methodologies emerging continuously.

This study has a clear objective. It aims to conduct a comprehensive review of augmented reality technology. The focus is on the technological advancements and the innovative challenges that come with it. This study will integrate the latest findings from various research fields. The goal is to provide medical professionals with a comprehensive perspective. This perspective will cover the application of AR technology in clinical practice. It will assess the maturity of existing technologies and guide practitioners on how to integrate these innovative tools into their surgical processes, thereby enhancing the precision and safety of surgeries.

## 2. Methods

### 2.1. Related Works

By integrating multiple technologies, AR-based surgical visual guidance systems can offer enhanced precision, more intuitive interactivity, and a higher degree of customization in the surgical experience. This not only increases the likelihood of successful surgical outcomes but also significantly improves patient safety. A prime example is Proprio, a company that has combined AI, computer vision, AR, and robotic technology to offer innovative 3D medical imaging and data management solutions for surgeries. Their technology assists surgeons in identifying surgical obstacles, planning operations, and sharing surgical data in real-time during the perioperative period [16]. Currently, Proprio has implemented pilot programs for neurosurgery and orthopedic surgery at institutions such as Seattle Children’s Hospital. Beyond the commercially available surgical guidance products that have been reported, exploring the potential of AR technology in surgical procedures is also a research topic for many scholars, which has given rise to numerous research prototypes. Table 1 lists some of the research prototypes published in recent years that utilize AR technology in perioperative surgical guidance. It is worth noting that precision, in the context of Augmented Reality (AR) technology, refers to the consistency and repeatability of measurements or computational outcomes. Within the realm of AR, precision pertains to the system’s ability to yield uniformly aligned image overlays and positioning information across various time points and conditions. A system with high precision can provide invariant image superposition and localization data in every operation, and it is capable of swiftly and accurately updating the position of virtual imagery in response to changes in the patient’s position during surgery, thereby maintaining congruence with the actual anatomical structures.

These technologies range from Optical See-Through Head-Mounted Displays (OST-HMDs) to AR navigation systems based on 3D displays, as well as advanced devices such as Microsoft HoloLens, and have shown significant potential in improving surgical accuracy. For instance, the system developed by Chen et al. achieved a high-accuracy 3D reconstruction with an average error of only 0.32 mm in minimally invasive knee surgery [19]. However, despite showing promise in laboratory settings, most of these technologies have not yet undergone clinical testing, which limits a comprehensive assessment of their performance in actual surgical environments. Moreover, while some studies provide specific accuracy data, such as the Target Registration Error (TRE) of 10.62 ± 5.90 mm in Ackermann’s research [18], overall, these technologies still require further validation and improvement in terms of accuracy and clinical applicability. Studies like the one conducted by Creighton et al. have demonstrated potential in the field of Total Shoulder Arthroplasty (TSA) using Microsoft HoloLens 1. These studies highlight the benefits of depth-sensing cameras. However, they lack clear accuracy values and clinical testing. As a result, it becomes difficult to evaluate their specific effectiveness in actual surgeries [20]. This is a limitation that needs to be addressed in future research. Therefore, to promote the transition of these promising technologies from the lab to the operating room, future research should focus on addressing the limitations of current technologies, such as improving accuracy, optimizing hardware performance, and enhancing system stability and user-friendliness.

In the academic community, experts generally affirm the advantages of applying Augmented Reality (AR) visual guidance systems during the perioperative period. However, despite this affirmation, these systems still face some challenges during their development that have not yet been fully resolved, which limit their effectiveness and accuracy in surgical navigation. Currently, the biggest challenge lies in improving the implementation accuracy of AR systems. Lin [17] and Liounakos [23] both emphasized the importance of refining AR systems from a technical standpoint to ensure their stability and consistency in clinical applications. Fida, in his research on integrating AR technology into the surgical process, summarized the direction of AR technology advancement into two main aspects: optimizing tracking patterns and accurately registering generated images with the real visual field [24]. This view has also been widely supported in other similar studies. In addition, hardware limitations, such as the fixed focal length issue of the HoloLens 1 and the insufficient performance of its depth-sensing camera, have been identified by Creighton [20] and others as the main factors causing errors. Therefore, continuous attention to and improvement of the integrated hardware systems used in the system are crucial for enhancing the application effects of AR technology in surgical navigation.

A rigorous analysis of the current literature and practical applications of perioperative AR visual guidance has revealed three critical challenges essential for enhancing the performance of these systems.

Challenge 1: image processing and 3D reconstruction proficiency: the accurate extraction and reconstruction of patient anatomy from preoperative imaging require further advancements in image processing and 3D reconstruction technologies. This includes the deployment of advanced deep learning models, such as U-Net and DeepLab, to segment organs, tissues, and blood vessels from volumetric imaging data like CT and MRI. These models are crucial for providing the anatomical models necessary for AR. The primary challenge is to increase the accuracy and processing speed of these models to enable the real-time delivery of precise 3D reconstructions during surgical procedures.

Challenge 2: seamless integration of integrated hardware systems: integrated hardware systems must overcome the challenge of integrating seamlessly with existing surgical processes. This necessitates the development of more efficient and stable technical solutions to achieve a synergistic operation between hardware devices and surgical navigation software. An AR surgical navigation system comprises three core components: virtual image or environment modeling, registration of the virtual environment with real space, and display technology that combines the virtual and real environments. The challenge is to ensure that these components function stably and accurately throughout surgical procedures, in harmony with the operating room’s workflow.

Challenge 3: precise spatial localization and registration technology: precise spatial localization and registration technologies are fundamental to achieving high-precision surgical navigation. These technologies, which link the virtual scene with the real scene through 3D registration techniques, anchor the virtual scene to the real scene’s coordinate system, ensuring a shared spatial context between the virtual and real environments. The challenge is to improve the accuracy and stability of registration and to reduce any latency or jitter that may be perceived by users during surgical interventions.

The use of AR technology has received notable attention in the medical field in recent years. Jud et al. conducted a systematic review in which they summarized the applicability of AR technology in orthopedic surgery, including instrument/implant placement, osteotomy, oncologic surgery, trauma surgery, and surgical training and education, and highlighted the potential of AR technology to improve surgical accuracy and reduce radiation exposure [25]. Kim et al. focused on the use of VR and AR technologies in orthopedic surgery, covering preoperative planning, surgical navigation, and training, and discussed how these technologies can improve surgical accuracy and patient satisfaction with appearance by simulating the surgical process [26]. In addition, a review study by Barcali et al. systematically analyzed literature published between 2019 and 2022, focusing on the use of AR technology in orthopedic, maxillofacial, and oncology surgery and evaluating the main solutions for AR technology, including the Microsoft HoloLens optical viewer and marker-based tracking and registration methods [27]. Currently, studies such as these are mainly dedicated to demonstrating the positive applications of AR technology in healthcare, but they also mention the need for technological integration, operational challenges and user experience improvements. However, review studies mainly devoted to the challenges faced by AR technology in the perioperative period with current state-of-the-art technological solutions, with a view towards providing valuable insights and directions for improvement in future research and clinical practice, have not yet been retrieved.

**Figure 1:**
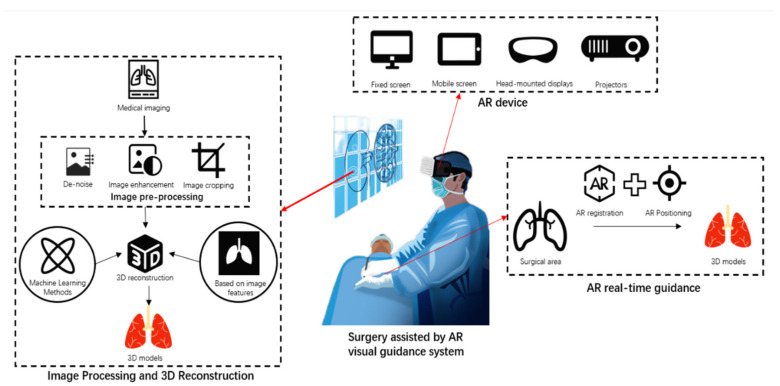
Technical components of the AR vision system.

The contribution of this study lies in its comprehensive review of the latest advancements in AR technology in the fields of surgical planning, navigation, and education, and it goes further by proposing innovative approaches to tackle technological challenges. Compared to the existing literature, this research delves deeper into how cutting-edge technologies like machine learning algorithms and skin electronic devices can enhance the accuracy and stability of AR visual guidance systems in clinical applications. Additionally, it offers unique insights into future technological trends, including the integration of AR with artificial intelligence, improvements in real-time tracking technologies, and the potential of skin electronic devices to increase the comfort and precision of surgical procedures. These discussions not only provide valuable information resources for medical professionals but also guide technology developers in setting future research directions, collectively promoting the advancement of surgical navigation technology to new heights. Figure 1 illustrates the technical composition of the AR visual system.

### 2.2. Literature Search Strategy

We selected papers published between 2014 and 2024 from the Web of Science, PubMed, and IEEE Xplore databases. Utilizing four sets of search strings, we conducted our search across titles, keywords, and abstracts, culminating in the inclusion of 193 studies:

1. First set: ((“AR *” or “Hybrid Reality *” or “Augmented Reality ”) and (“surgery” or “surgical operation *” or “perioperative period *” or “preoperative planning *”) and (“visual guidance *” or “visual direct *” or “visual lead *”));
2. Second set: ((“AR *” or “hybrid reality *” or “Augmented Reality ”) and (“medicine” or “medical field *” or “medical Science *”) and (“3D reconstruction *” or “3D modeling *”));
3. Third set: ((“AR *” or “hybrid reality *” or “Augmented Reality *”) and((“spatial positioning *” or “spatial navigation *” or “registration *”)));
4. Fourth set: ((“AR *” or “Hybrid Reality *” or “Augmented Reality *”) and (“display device *” or “display screen *” or “HMD *”)).

**Figure 2:**
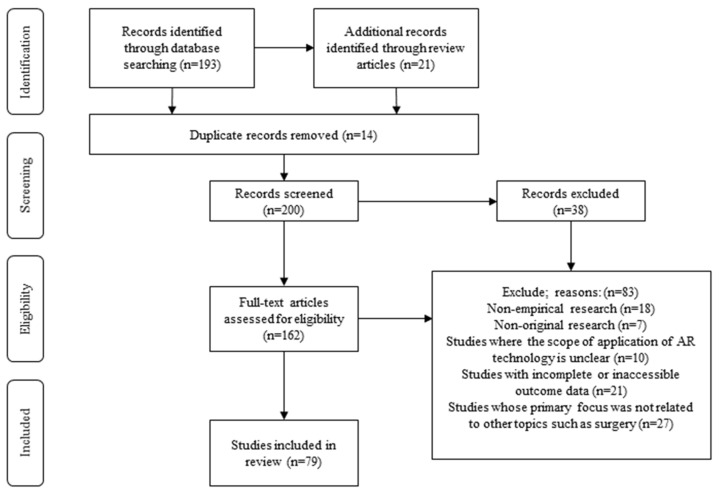
Search and screening process.

Furthermore, among these 193 articles, 7 were review articles. We employed the aforementioned search strings to sift through the reference lists of these seven reviews, which yielded an additional 21 papers. By examining the abstracts of these papers, we initially eliminated duplicates. Subsequently, through a full-text review, we further excluded papers that met one or more of the following criteria: non-empirical studies; non-original research; studies with unclear applications of AR technology; studies with incomplete or inaccessible result data; and studies primarily focusing on themes unrelated to surgical operations. Ultimately, we selected 79 articles that serve as the principal sources of information for this paper, as illustrated in Figure 2.

## 3. Image Processing and 3D Reconstruction for AR Visual Guidance Systems

Before surgical procedures, basic anatomical structures can be identified through medical imaging, typically Computed Tomography (CT) [28] or Magnetic Resonance Imaging (MRI) [29]. Although these images contain all the necessary information about tumors, major blood vessels, and the tissue environment, surgeons may find it difficult to perceive the relationships between these structures during surgical planning and execution. Therefore, providing surgeons with tools that can simplify the interpretation of traditional images seems crucial. Among these tools, three-dimensional (3D) visualization shows significant advantages compared to the standard 2D slice visualization [30,31], which is also the fundamental reason why many studies introduce AR as a solution. The realization of AR effects is based on two main processes: the 3D modeling and visualization of anatomical or pathological structures appearing in medical images and the deployment of this visualization onto the actual patient. Among them, medical image reconstruction is the most basic and important technical element in building an AR visual guidance system [32].

The evolution of 3D reconstruction techniques grounded in medical imaging harks back to the 1970s, with early scholars pioneering the reconstruction of 3D models from sequential two-dimensional images through ray tracing technology [33]. As computer graphics and vision technologies have matured, diverse 3D reconstruction methodologies have surfaced, encompassing voxel-based, surface-based, and regularization-based approaches [34]. The dawn of the 21st century witnessed a surge in the sophistication of medical imaging equipment, which in turn accelerated the evolution of 3D reconstruction technologies. Today, such technologies, leveraging CT and MRI, are instrumental in diagnosing a spectrum of conditions, including tumors and cardiovascular diseases [35]. The utility of 3D reconstruction has expanded into realms such as virtual surgical simulations, surgical planning, and postoperative evaluations [36]. The embrace of AR-based visual guidance in the perioperative period by the clinical community is significantly attributed to the transformative improvements in 3D reconstruction outcomes facilitated by machine learning technologies. Handling vast and intricate datasets to identify useful patterns and features has long posed a formidable challenge for traditional computational methods. Machine learning, particularly deep learning, has risen to the forefront with its ability to automate the extraction of features from 2D imaging data and construct accurate 3D models using algorithms such as Convolutional Neural Networks (CNNs) and Generative Adversarial Networks (GANs). Moreover, machine learning technologies possess the innate capability to continually learn from new data, thereby optimizing the reconstruction process and enhancing the accuracy and reliability of the models. As the volume of medical imaging data continues to grow, the application of machine learning is demonstrating immense potential and value in managing this data, reducing noise, improving image resolution, and automating clinical workflows. Consequently, the integration of machine learning techniques into the realm of 3D medical imaging reconstruction not only elevates the quality of diagnosis and treatment but also propels the advancement of personalized and precision medicine. For example, CNNs are adept at automatically extracting features from CT and MRI images to accomplish 3D reconstruction [37]. Furthermore, innovative reconstruction methods leveraging embeddings have emerged, offering a novel angle by converting images into textual data and reconstructing 3D models with the aid of embeddings [38]. Table 2 encapsulates a compendium of recent scholarly milestones in harnessing machine learning for the 3D reconstruction of medical imagery. Accuracy, in the context of AR technology, denotes the degree to which virtual imagery or data corresponds to the patient’s actual anatomical structures. A system possessing high accuracy guarantees that the virtual information is precisely overlaid on the corresponding real-world locations, devoid of any systemic deviation.

In the realm of 3D reconstruction, the application of machine learning methods, particularly deep learning algorithms, has achieved remarkable breakthroughs, especially in the field of medical imaging technology. For instance, in a 2024 study by Prakash et al., a Conditional Generative Adversarial Network (cGAN) was utilized to distinguish between tumor and non-tumor tissues in CT scan images, achieving a diagnostic accuracy rate of 96.5% [39]. This significant outcome not only demonstrates the immense potential of deep learning in enhancing the precision of clinical diagnostics but also reflects its exceptional capability in handling complex medical data. Similarly, in 2023, Zi et al. employed a U-Net architecture in the Brain Tumor Segmentation Challenge (BraTS), obtaining a Dice Coefficient of 85.3% and an Intersection over Union (IoU) of 78.9% [40]. These accomplishments further confirm the effectiveness and prospective application of deep learning networks in image segmentation tasks.

In the realm of feature extraction, the Lineformer model, proposed by Cai et al. captures the internal structure of objects by simulating the interdependencies within X-ray line segments, achieving significant performance enhancements in novel view synthesis and CT reconstruction tasks compared to existing NeRF-based methods [42]. The innovation of this model lies in its ability to address the sparsity of X-ray images by focusing on the spatial relationships between line segments, which is a relatively novel approach in medical image analysis. Shen et al. utilized Recurrent Neural Networks (RNNs) to extract nonlinear features from CT images, achieving an average reconstruction accuracy of 62.9% based on the Structural Similarity Index (SSIM) [43]. This result highlights the reliability of deep learning in processing complex medical data and extracting deep-level features. The use of RNNs demonstrates strong modeling capabilities for time-dependent sequences, which is crucial for handling continuous medical image data. In 2023, Hong et al. combined Generative Adversarial Networks (GANs) with Long Short-Term Memory networks (LSTMs) for the reconstruction of lung tumors, showcasing the superiority of their method through Hamming and Euclidean distance metrics [44]. This combination of generative and sequential models not only produces high-quality images but also optimizes the reconstruction process by leveraging the temporal information from LSTMs. Perdios et al., in 2019, focused on the reconstruction, recovery, and enhancement of ultrasound images, demonstrating that CNN-processed images could improve the performance of vector flow estimation in certain aspects [45]. This finding is of significant importance for enhancing the diagnostic value of ultrasound images.

Although these studies demonstrate the immense potential of machine learning in 3D reconstruction, several challenges remain. For instance, deep learning models typically require substantial computational resources, which may limit their application in resource-constrained environments. Additionally, model performance heavily relies on the quality and diversity of training data, and obtaining and annotating a large volume of training data for 3D reconstruction tasks is challenging. The generalizability, interpretability, and real-time performance of deep learning models are also critical issues in this field. To overcome these challenges, researchers are developing more efficient algorithms, such as the Weight Pruning U-Net (WP-UNet) proposed by Prakash et al. and the Deep Learning-based Iterative Reconstruction (DL-MBIR) strategy suggested by Ziabari et al., aiming to optimize computational efficiency and the practicality of model deployment [46]. These methods aim to reduce the computational resource requirements by decreasing the number of model parameters and improving parallel processing capabilities, while maintaining or enhancing model performance.

Looking to the future, breakthroughs in 3D reconstruction within the realm of machine learning are anticipated to manifest across various critical avenues. The enhancement of resource efficiency is becoming a top priority. Researchers are increasingly dedicated to creating more streamlined algorithms and network frameworks. These improvements aim to reduce the reliance on substantial computational resources. This reduction is particularly important for deep learning models during both the training and inferencing phases. By doing so, they hope to make these models more efficient and accessible. An exemplar approach involves the refinement of neural network architectures to sustain output excellence while concurrently curtailing the model’s parameter count, leading to a reduced need for storage and computational capabilities [47]. In tandem, the sphere of data acquisition and enhancement will garner increased research attention, with a spotlight on elevating the caliber and spectrum of training data. This could encompass the innovation of automated data annotation tools, the generation of synthetic data methodologies, and the investigation of data augmentation techniques, all aimed at fortifying model proficiency and flexibility in the face of varied conditions [48]. Aiming to augment model generalizability, upcoming research endeavors will concentrate on the conception of more resilient network architectures and the introduction of innovative training strategies, such as domain adaptation and meta-learning. These initiatives will facilitate machine learning models to adapt more seamlessly to a spectrum of datasets and environmental contexts, enhancing their steadfastness and dependability in real-world applications. For instance, domain adaptation techniques will enable models to transpose learning from a domain abundant with data to one that is data-sparse, while meta-learning will afford models the agility to swiftly adjust to novel tasks within the confines of limited data [49].

## 4. Spatial Positioning and Registration Techniques for AR Visual Guidance Systems

In 1947, a milestone in medical history was achieved at Temple University School of Medicine in Philadelphia, with the first stereotactic neurosurgery on the human brain conducted successfully. Spiegel detailed in his report how the team utilized plaster models alongside ventriculography to pinpoint surgical targets, marking a colossal leap forward in technology at that time [50]. This innovation significantly elevated the precision of neurosurgical procedures, minimized harm to adjacent healthy brain tissue, and bolstered the safety and efficacy of surgeries. Fast forward to the present, over seven decades later, surgical navigation systems have evolved exponentially beyond their rudimentary stereotactic predecessors. They now integrate preoperative imaging with real-time intraoperative visuals to construct highly accurate 3D models, offering physicians an AR-assisted framework for precise surgical routes and target localization [51,52,53,54]. These AR visual guidance systems harness foundational medical imaging data from ultrasound, X-rays, CT scans, and MRI to generate a patient-specific 3D model, which surgeons then employ to devise surgical strategies. Intraoperatively, these systems leverage real-time tracking to navigate the surgeon’s maneuvers and affirm the surgical plan’s execution. The crux of this technological marvel lies in the registration and positioning technologies that ensure the virtual imagery aligns precisely with the patient’s anatomical structures.

The act of registration is the alignment of virtual models or images with a patient’s actual anatomical structures, a process that is typically completed prior to the start of surgery. Its purpose is to create a mapping that enables the virtual images in the surgical navigation system to overlay precisely onto the patient’s anatomical reality [55]. Manual registration stands as the most direct approach, where surgeons are tasked with manually adjusting the virtual model to correspond with the patient’s anatomy during the procedure. In situations where advanced equipment is not available or is hard to reach, manual registration can be a practical solution. It is particularly useful for simple surgical navigation tasks, like minor outpatient procedures. Additionally, manual registration is beneficial in environments with limited resources [56]. This approach ensures that basic navigation needs can still be met even under constrained conditions. Gregory et al. employed manual registration in reverse shoulder arthroplasty to match the visible bone segments with their holographic counterparts, effectively addressing the clinical challenge of limited bone volume in the dome area [57]. Li et al. utilized manual registration to align a catheter with a holographic trajectory during the insertion of an external ventricular drain (EVD), thereby completing the registration process [58]. However, the precision of manual registration can fluctuate considerably due to the surgeon’s proficiency or visual obstructions in the operating field, presenting a critical risk in surgical contexts and explaining its limited widespread adoption [55]. Azimi et al. introduced a novel registration technique using a black-box method for HMD calibration, with tracker data as inputs and the 3D coordinates of virtual objects in the observer’s visual field as outputs. This technique has refined the average re-projection error of manual registration to 4 mm, which could potentially augment the practicality of manual registration in surgical guidance [59]. Yet, in an array of clinical settings, automatic registration methods leveraging sensing technologies or machine vision are more commonly preferred. The choice of feature extraction in these automatic methods accommodates a variety of patient anatomies, surgical equipment, and procedural nuances. Currently, there are three predominant medical image registration techniques: point-based, surface-based, and marker-based registration. Point-based registration focuses on identifying and correlating specific points between preoperative imaging and actual anatomical structures, making it suitable for surgeries requiring precise localization of small anatomical points. Surface-based registration utilizes 3D surface models to match the patient’s anatomical surfaces, ideal for procedures where distinct surface features are present, such as cranial reconstructions. Marker-based registration, on the other hand, relies on placing known geometric markers on the patient and recognizing these markers in preoperative images to align the images with the anatomical structures, necessitating high precision in marker placement and recognition. Each technique has its unique applications and limitations, and selecting the appropriate registration method is crucial for enhancing the precision of surgical navigation. Table 3 compiles the most recent studies on diverse registration techniques, delving into the selection of application contexts and the attainment of precision benchmarks

In the recent domain of medical imaging and computer-assisted surgery, research on registration methods has emerged as a topical subject. Registration techniques play an indispensable role in AR navigation systems, particularly in surgical environments that demand high precision, such as pediatric tumor surgery and neurosurgery. From the provided literature, various registration methods can be observed, including marker-based, markerless, surface-based, and volume-based approaches.

In the study by Souzaki et al., an AR navigation system was developed, leveraging preoperative CT and MRI imaging for endoscopic surgery in pediatric tumors [61]. This system employs an optical tracking system to align the reconstructed 3D images with surface markers during surgery, achieving precise superposition of virtual imagery onto actual anatomical structures. The key to this method lies in its adaptability to patient movement, ensuring the alignment of virtual information with real structures, thereby enhancing surgical accuracy and safety. On the other hand, Yavas et al. explored an AR neuronavigation system based on 3D-printed markers, which utilizes mobile devices to recognize specifically designed markers on the patient’s skull, providing 3D imaging [67]. The innovation of this method is its provision of a cost-effective, user-friendly, and highly accurate navigation technique, reducing reliance on high-cost navigation systems and shortening the time required for preoperative image registration. Goerres et al. investigated a planning, guidance, and quality assurance system for pelvic screw placement based on deformable image registration [62]. This system incorporates automatic planning of pelvic screw trajectories and accounts for the deformation of surgical devices, such as K-wire deflection. By performing 3D–2D image registration between preoperative CT and intraoperative fluoroscopy, it achieves precise positioning of surgical instruments. Joeres et al. proposed a two-step registration process to address the time pressure encountered during the repair of resection sites in laparoscopic surgery [63]. The method involves an initial accurate registration before tumor resection and a rapid re-registration using artificial markers after resection. Validated in a simulated use study, this approach demonstrated faster registration speeds compared to traditional anatomical landmark-based registration and improved accuracy when the primary registration was successful.

In summary, these studies showcase the potential of registration methods in enhancing the precision and efficiency of surgical navigation. Although each method has its strengths and limitations, they collectively contribute to the advancement towards more accurate, faster, and user-friendly surgical navigation systems.

The versatility of registration technologies has immensely expanded the horizons for AR visual guidance, a technique that has garnered widespread acclaim for its contributions to surgical precision, safety, and efficiency. Given that a patient’s position can vary throughout a surgical procedure, the incorporation of accurate positioning technology is essential for ensuring the precise overlay of virtual information onto actual anatomical structures [69]. Positioning technology encompasses the real-time tracking and computation of the location and orientation of surgical instruments or the patient’s anatomy during surgery. This ensures that virtual imagery remains in precise alignment with the physical structures, even in the event of patient movement or tissue distortion [70,71]. The AR visual guidance system, through its dynamic real-time tracking capabilities, can adjust the virtual models to accommodate any anatomical changes, thereby offering surgeons an unerringly accurate surgical perspective.

Developers crafting AR applications often prioritize the most straightforward self-localization methods. These methods harness the cameras embedded in AR devices to detect environmental changes, ensuring that the superimposed virtual 3D imagery aligns with the relative positions of the actual surroundings [72]. Within this spectrum of technologies, the HMD stands as the go-to device for self-localization. It facilitates a smooth transition between virtual and real-world patient anatomy through holographic optics and manual alignment techniques, enabling markerless, natural tracking [73,74]. For example, Scherl et al. deployed the HoloLens^®^1 (Microsoft Corporation, Redmond, WA, USA) to offer preoperative planning for surgeries in the parotid gland area [75]. Their approach necessitated the manual alignment of 2D and 3D augmented reality models derived from MRI scans with the patient’s physical form. Creighton et al. likewise utilized HMD technology to aid in orthopedic procedures, affirming the viability of this method [20]. Despite the high degree of system integration and user-friendliness offered by automatic localization technologies, concerns have been raised by some researchers regarding the potential inaccuracies in the manual alignment of holographic images with actual anatomical structures [76,77].

To tackle this challenge, the academic sphere has embraced sensor technology and machine vision as innovative approaches. Fischer et al. integrated an infrared camera system with a machine vision algorithm to enhance positioning precision through the tracking of distinctive feature points [78]. Schwald et al. investigated the combined effect of an optical tracking setup, consisting of a stereo camera system with infrared filters and frame grabbers, alongside the pciBIRD sensor, in the context of AR localization [79]. In a more groundbreaking development, Racadio et al. introduced a novel camera-free eye-tracking sensor designed for AR glasses. Leveraging laser scanning technology, this sensor employs a Micro-Electro-Mechanical Systems (MEMSs) micromirror to direct an infrared laser beam, reflected in the eye area, with the scattered light detected by a photodiode [80]. Technically, eye-tracking technology enhances the interactivity, user experience, and seamless alignment of visual content in Augmented Reality (AR) systems by monitoring users’ line of sight in real time, thereby achieving a more intuitive and personalized superposition of virtual images. This method offers not only high integration but also the benefit of low power consumption compared to traditional video-oculography (VOG) systems. Sylvain et al. propelled the evolution of AR systems by enabling the automatic localization of endoscopes within intraoperative CT imagery [81]. This advancement renders the AR system independent of external tracking systems or the need for endoscopic image analysis, offering a more intuitive and accurate navigation solution for laparoscopic surgeries.

As registration and positioning technologies advance, AR visual guidance systems are making welcome strides towards a broader application horizon in the medical field. A multitude of studies have validated the clinical viability of AR systems, marking a transition from the pressing needs of surgeons to a “golden era” of recognized value. Particularly in the challenging realm of vascular surgery, the demand for precision in minimally invasive vascular interventions has reached sub-millimeter precision [82], while the accuracy of AR systems in registration and positioning is still assessed in millimeters. The further amalgamation of machine learning technologies foretells a breakthrough in the precision of AR visual systems, charting a new course for future advancements. Pioneering research has already attested to the vast potential of these integrated technologies. For instance, Fu et al. introduced a non-rigid magnetic resonance–transrectal ultrasound (MR-TRUS) image registration framework for prostate interventions [83]. This framework utilizes a two-pronged approach for image analysis. First, it employs convolutional neural networks (CNNs) to segment the prostate in both magnetic resonance (MR) and transrectal ultrasound (TRUS) images. Second, it integrates a point cloud-based network to facilitate rapid 3D point cloud matching. This combination of technologies has achieved a mean surface distance (MSD) of 0.90 ± 0.23 mm, demonstrating high precision in aligning the segmented images. In a concurrent development, Elgarba et al. leveraged artificial intelligence (AI) to automate the registration of Cone Beam Computed Tomography (CBCT) [84]. They conducted a study involving six intraoral scan-cone beam computed tomography (IOS-CBCT) scans to evaluate consistency. The study demonstrated that the AI-driven IOS and the registration of artifact-rich CBCT images were reliable and efficient. Furthermore, these registrations were expertly accurate and highly consistent, marking a significant advancement in the field. The pursuit of enhancing the precision of AR visual system registration and positioning to even higher echelons, while also achieving notable advancements in real-time capabilities, multi-modal integration, intelligent diagnostics, and full perioperative integration, will undoubtedly require our collective endeavor.

This section provides a comprehensive discussion on the registration and positioning challenges encountered in AR-based surgical visual guidance systems. Initially, the manual registration process is not only time-consuming but also highly dependent on the individual skills of the surgeon, which may lead to inaccurate navigation in emergency or complex surgical situations. Furthermore, existing hardware devices, such as the HoloLens 1, suffer from limitations in fixed focal length and depth-sensing camera performance, impacting the precision of AR systems. Additionally, while automatic registration techniques offer greater accuracy and consistency, they still face technical challenges in adapting to different patient anatomies and surgical equipment. Concurrently, the need for AR systems to update the position of virtual models in real-time during surgery, due to patient movement, poses higher demands on real-time tracking technologies.

To address these challenges, current research has proposed a range of solutions. These include improving manual registration techniques, such as the black-box method proposed by Azimi et al., to reduce errors and enhance the accuracy of surgical navigation [59]. Concurrently, efforts are being made to optimize hardware systems by developing cameras with higher resolution and superior depth perception capabilities, thereby enhancing the overall performance of AR systems. The exploration of advanced sensor technologies and machine vision algorithms for automatic registration is also gaining popularity, like the infrared camera system by Fischer et al., aiming to improve the accuracy and efficiency of registration [78]. Moreover, the integration of more sophisticated real-time tracking technologies, such as the MEMSs micromirror technology by Racadio et al., will achieve more accurate real-time tracking, further enhancing the accuracy of surgical navigation [80].

## 5. Status of AR Device Applications

The journey of AR technology has been one of significant evolution, from the basic overlaying of images to the sophisticated interactive experiences we enjoy today. Contemporary AR devices offer not only lifelike visual effects but also enable naturalistic interaction through gesture recognition and voice control. These features empower users to engage with virtual content in ways that are more intuitive and seamlessly integrated with their natural behaviors.

In the realm of surgical procedures, AR visual guidance systems are broadly categorized into two primary types: intelligent glasses and hybrid reality displays. These cutting-edge devices significantly augment the work of surgeons by offering real-time visual feedback and precise surgical navigation. HMDs, in particular, are emerging as the leading technology in this domain, favored for their portability, utility, and human–computer interaction capabilities. Smith’s research emphasizes the significance of tailoring HMDs for medical use [85]. It highlights several key features that are essential for such applications. These include lightweight construction, which is crucial for comfort. High transmittance rates are also important for clear image display. Prolonged battery life ensures the devices can be used for extended periods. Doughty et al. have provided further evidence supporting the superiority and efficacy of Optical See-Through Head-Mounted Displays (OST-HMDs) in surgical settings. Their research offers valuable insights into the benefits of these devices. As a result, there is a growing trend in both research and practice to use OST-HMDs like Microsoft HoloLens. This trend also explores interactive modalities such as hand gestures and voice commands, enhancing the usability of these devices in surgical contexts [86]. Despite this, projection display technologies and mobile video display units continue to hold relevance in certain specialized applications. Table 4 encapsulates a comprehensive review of the display devices currently in circulation, alongside an assessment of their strengths and limitations.

The enthusiasm with which doctors embrace the integration of AR technology in neurosurgical navigation comes as no great shock [97]. AR technology, by overlaying virtual information onto the patient’s genuine anatomical structures, substantially amplifies the precision and security of surgical operations. The real-time enhancement of visual data not only streamlines the surgical workflow but also bestows upon surgeons a perspective and depth of understanding that were previously unattainable. Yet, while AR technology holds vast theoretical promise, the practical implementation encounters certain hurdles. Notably, the discomfort and strain that head-mounted display devices (HMDs) may impose on physicians during extended surgical sessions have been validated by a plethora of research [98,99]. The potential for neck and head fatigue from prolonged use could compromise surgical efficiency and the surgeon’s focus.

Addressing this challenge, recent studies have introduced a groundbreaking solution: skin electronics [100]. These devices, characterized by their thinness and flexibility, offer a nearly unnoticeable wearing experience that alleviates the discomfort physicians may encounter during surgery. Skin electronics devices address the discomfort challenges posed by HMDs through a variety of innovative approaches. With their thin, flexible design, these devices significantly alleviate the physical strain associated with prolonged wear, particularly around the head and neck. Compared to traditional HMDs, the unobtrusive nature of skin-electronics allows for seamless integration into wearable patches or smart clothing, offering users a more natural and comfortable experience. They also provide direct haptic feedback to the skin, simulating real touch sensations, which reduces reliance on visual displays and enables interaction with AR content without the need for an HMD. Moreover, these devices can be tailored to fit the user’s body shape and movements, ensuring a comfortable fit and greater freedom of movement while minimizing physical constraints. Skin electronics devices also help reduce eye strain, potentially eliminating the need for users to continuously focus on the small displays found in HMDs. Furthermore, skin electronic devices come equipped with sophisticated input and output capabilities, facilitating superior signal acquisition and responsive feedback [101]. For example, physicians can monitor patients’ vital signs non-invasively through skin electronics, including heart rate, blood pressure, and muscle activity—information that is pivotal for making informed, real-time surgical decisions. Additionally, these devices are capable of delivering haptic feedback, enhancing the tactile sensation of surgical instrument manipulation for the surgeon [102,103]. Skin electronics have become self-sufficient with the integration of independent power sources. This integration eliminates the need for external power supplies and the hassle of tangled wiring. As a result, the convenience and safety of surgical operations are significantly enhanced. The integrated system, powered by efficient data processing and intelligent algorithms, is adept at providing real-time, customized surgical assistance tailored to the surgeon’s specific needs.

With the relentless march of technological progress, there is ample justification for our belief that skin electronic devices are poised to assume a more pivotal role in the medical field of the future. These devices hold the promise of not only mitigating the existing challenges associated with AR visual guidance systems but also of forging groundbreaking surgical techniques and therapeutic approaches. They are set to deliver a treatment experience that is characterized by enhanced precision and safety for patients. Ongoing research endeavors will delve into the untapped potential of these devices across diverse healthcare settings and will be dedicated to refining their capabilities and elevating the user experience to unprecedented levels.

## 6. Conclusions

This paper offers an exhaustive retrospective on the evolution, technical delineations, and clinical exemplars of AR technology within the realm of perioperative visual guidance. It also delves into an analysis of the existing challenges and prospective trends on the horizon. AR technology, distinguished by its capacity to merge virtual information with the tangible anatomical structures of patients, has markedly enhanced the precision, safety, and efficacy of surgical interventions. The application of AR in surgery has evolved significantly. Initially, it began with simple visualization systems. Today, it has advanced to incorporate sophisticated technologies such as artificial intelligence, computer vision, and robotics. This evolution has demonstrated the immense potential and substantial value of AR in surgical procedures. This technology stands as a testament to the transformative impact of innovative solutions on the field of surgery, promising to shape the future of medical procedures with its ceaseless evolution.

In the evolution of AR surgical visual guidance technology, researchers have encountered numerous challenges, including enhancing system implementation precision, overcoming hardware limitations, optimizing automatic registration techniques, strengthening real-time tracking capabilities, improving the generalizability and interpretability of models, enhancing the comfort and practicality of devices, and achieving multimodal integration and intelligent diagnostics. A variety of innovative solutions have risen to meet these challenges. Although these solutions have not been fully implemented in the medical field, they lay a solid groundwork for technological progress. Specifically, they hold great potential for advancing surgical visual guidance systems.

These solutions can be summarized as follows: improving system precision through technical optimization and hardware upgrades; refining the automatic registration process with sensor fusion and machine learning algorithms; enhancing real-time tracking capabilities with advanced sensors and algorithmic optimizations; bolstering model generalizability and interpretability through multicenter data training and model explanation tools; developing thin and flexible skin electronic devices and ergonomic designs to enhance device comfort and practicality; and constructing platforms that integrate various imaging data and diagnostic information, utilizing AI technology to provide intelligent diagnostic support. These comprehensive strategies not only promote the application of AR technology in surgical navigation but also lay a solid foundation for the future development of the medical field, heralding a significant enhancement in surgical precision and safety.

As we gaze into the future, the evolution of innovative technologies like skin electronics heralds a seamless integration of AR into medical practice, promising surgical assistance that is more comfortable, intuitive, and precise than ever before. With the profound amalgamation of machine learning, breakthroughs in the precision of AR visual systems are on the horizon. We eagerly anticipate notable progress in real-time capabilities, multi-modality integration, and intelligent diagnostics of AR technology, enabling comprehensive access throughout the perioperative period. This progression is set to not only accelerate the advancement of surgical navigation systems but also to deliver treatment experiences that are more accurate and secure for patients, heralding a new “golden era” in healthcare.

## Figures and Tables

**Table 1:** Selected prototype studies on the use of AR technology in perioperative surgical guidance.

Reference	Year	Technical Solution	Fields of Use	Achieved Precision	Are Clinical Tests Included?
Lin et al. [17]	2018	Optical See-Through Head-Mounted Display (OST-HMD) for image-guided percutaneous spine procedures	Percutaneous Spine Procedures	Comparable to traditional monitor in terms of procedural time and dosimetry	No
Ackermann et al. [18]	2021	AR navigation system with HMD, overlaying Computed Tomography (CT) data using fiducial markers	Lateral Skull Base Surgery	Target Registration Error (TRE) of 10.62 ± 5.90 mm	No
Chen et al. [19]	2021	AR navigation system with 3D display, tissue properties-based deformation method	Minimally Invasive Knee Surgery	Mean error of 0.32 mm for virtual arthroscopic images	No
Creighton et al. [20]	2020	Image-based AR guidance using HMD for Total Shoulder Arthroplasty (TSA)	TSA	Not explicitly provided; depth sensing camera performance identified as a major error source	No
Deib et al. [21]	2018	Performing image-guided spinal interventional surgeries using OST-HMD	Needle Placement Procedures	Significantly reduced placement errors with shape display compared to rigid needle assumption	No
Gu et al. [22]	2021	Head-up display-assisted endoscopic lumbar discectomy	Lumbar Discectomy	-	No
Liounakos et al. [23]	2020	Endoscopic Lumbar discectomy assisted by HMD	Ganz Periacetabular Osteotomy (PAO)	Osteotomy starting points error of 10.8 mm	No

**Table 2:** Recent research on 3D reconstruction using machine learning method.

Usage Scenarios	Reference	Year	Machine Learning Methods	Application Areas	Achievement Accuracy
Image segmentation	Prakash et al. [39]	2024	Conditional Generative Adversarial Network (cGan)	Correctly distinguishing tumor from non-tumor tissue in CT scans	The diagnostic accuracy has increased to 96.5%
	Zi et al. [40]	2023	U-Net Architecture	Brain Tumor Segmentation Challenge (BraTS)	Dice Coefficient = 85.3%, Intersection over Union (IoU) = 78.9%
	Cheng et al. [41]	2018	Deep Neural Network (DNN)	Used for image denoising and super-resolution	-
Feature extraction	Cai et al. [42]	2024	Line Segment-based Transformer (Lineformer)	Capturing the internal structure of objects by simulating the dependencies within each segment of X-rays	SAX-NeRF achieves 12.56 dB and 2.49 dB improvement over existing NeRF-based methods on new view synthesis and CT reconstruction tasks, respectively
	Shen et al. [43]	2019	Recurrent Neural Network (RNN)	Capturing non-linear features from CT images	Average reconstruction accuracy of 62.9% based on Structural Similarity Index (SSIM)
Model reconstruction	Hong et al. [44]	2023	Combining Generative Adversarial Networks (GAN) and Long Short-Term Memory Networks (LSTM)	Lung tumor reconstruction	The method shows superiority on Hamming and Euclidean distance metrics
	Perdios et al. [45]	2019	CNN	For reconstruction, recovery, and enhancement of ultrasound images	CNN-processed images improve the performance of vector flow estimation in some ways
Efficiency optimization	Prakash et al. [39]	2024	Weight Pruning U-Net (WP-UNet)	Optimizing computational efficiency	-
	Ziabari et al. [46]	2018	Deep Learning Model Based Iterative Reconstruction (DL-MBIR)	A strategy for multi-GPU implementation is proposed	-

**Table 3:** Typical studies using different registration methods.

Reference	Year	Registration Method	Application Scenario	Achievement Precision	Limitations
Liang et al. [60]	2016	Point-based Registration	Radioactive seed implantation for prostate cancer	0.44 ± 0.07 mm	Electromagnetic localizer susceptible to interference
Souzaki et al. [61]	2013	Point-based Registration	Endoscopic surgery for pediatric tumors	Precision has met surgical requirements	Problems of movement and deformation of organs during surgery
Goerres et al. [62]	2017	Point-based Registration	Percutaneous screw fixation of pelvic fractures	Within 1.1 mm	Geometric errors introduced by deformation of surgical instruments
Joeres et al. [63]	2021	Surface-based Registration	Laparoscopic tumor resection site repair surgery	Average target registration error (TRE) increased by an average of 2.35 mm	Clinical applicability yet to be demonstrated
Han et al. [64]	2021	Surface-based Registration	Dental surgery	Mean lateral biases in tooth surface registration are clinically acceptable	Not suitable for patients with edentulous jaws or few remaining teeth
Hu et al. [65]	2021	Surface-based Registration	Assisted femoral drilling	4.90 ± 1.04 mm in video perspective (VST); 4.36 ± 0.80 mm in optical perspective (OST)	Must “anchor” strategy to solve occlusion problems
Shao et al. [66]	2022	Marker-based Registration	Aids in surgical planning, medical training, and surgical procedures	-	Stability and precision issues in different light and environments
Yavas et al. [67]	2021	Marker-based Registration	Neurosurgery	Average positioning error 1.70 ± 1.02 mm	Brain displacement or deformation due to cerebrospinal fluid leakage or surgical location
Figueira et al. [68]	2022	Marker-based Registration	Surgical navigation	Average fusion error 0.70 ± 0.16 mm	Image marker may be obscured during the procedure

**Table 4:** AR display devices for intraoperative visual guidance.

Display Device Classification	Reference	Year	Application Field	Advantages	Disadvantages
Fixed video display	Kawakami et al. [87]	2024	Dental surgery	High-resolution display	Requires frequent diversions from the doctor
	Huang et al. [88]	2024	Dermatological surgery		
Mobile video display	Mehta et al. [89]	2024	Cardiovascular surgery	Adaptation to complex surgical environments	Screen size limits implementation; screen stabilization issues
	Dogan et al. [90]	2024	Craniotomy		
Translucent screen	Choi et al. [91]	2024	-	Intuitive; in situ magnification	Limited perspective
HMD	Judy et al. [92]	2024	Spinal surgery	Immersive experience	Burden of surgery
	Verhellen et al. [93]	2024	Orthopedic surgery		
	Kann et al. [94]	2024	Thoracolumbar spine trauma surgery		
Projection display technology	Ibrahim et al. [95]	2024	Facial surgery	Large space for surgical operations	Distortion problems; lower image resolution
	Mamone et al. [96]	2020	Osteotomy		
